# A Mass Spectrometric Approach to the Proteomic Profiling of the *Canis lupus familiaris* Acquired Enamel Pellicle on Hydroxyapatite Discs

**DOI:** 10.1177/08987564221097188

**Published:** 2022-05-12

**Authors:** Melissa M. Grant, Sabah Pasha, Taichi Inui, Iain Chapple, Steve Harris, Lucy Holcombe

**Affiliations:** 1 School of Dentistry, 1724 University of Birmingham, Birmingham, UK; 2 41854 WALTHAM Petcare Science Institute, Melton Mowbray, UK

**Keywords:** adsorption, canine, dog, enamel pellicle, saliva, mass spectrometry

## Abstract

The acquired enamel pellicle (AEP) is a multi-protein film attached to the surface of teeth, which functions to lubricate the dental surface, form an anti-erosive barrier and exhibits antimicrobial properties. The initiation of AEP formation occurs within seconds of exposure to saliva, a biofluid rich in protein species. While there have been many publications on the formation of human AEP there is little research on the composition of canine AEP during its acquisition. The aim of these studies was to explore the composition of canine AEP formation, utilising hydroxyapatite (HA) discs as a tooth substitute matrix, over time. Qualitative and quantitative proteomics techniques using tandem mass tag labelled peptides and LC-MS/MS were used to follow the formation of canine AEP on hydroxyapatite discs over the course of an hour. Proteins adsorbed to the HA surface included highly abundant proteins in canine saliva, antimicrobial proteins, protease inhibitors and the buffering agent carbonic anhydrase. Greater understanding of the canine AEP deepens fundamental knowledge of the early processes driving bacterial colonisation of the tooth surface and subsequent plaque accumulation.

## Introduction

The acquired enamel pellicle (AEP) is a thin acellular film of proteins or glycoproteins selectively adsorbed to the surface of the teeth after exposure to saliva. The AEP has a number of functions: it is utilised by microbes in the oral cavity to colonize the tooth surface through protein-protein interactions and carbohydrate binding; it acts as a semi-permeable barrier regulating demineralization-mineralization processes at the tooth surface; and it can prevent abrasion by working as a lubricative film. The proteins may be present as intact proteins or modified/cleaved forms.^1^ Many techniques have been employed to identify and explore the protein composition of human AEP, which range from amino acid profiling *in vitro*^2^ and *in vivo*,^3^ to structural studies by electron microscopy^4^ and atomic force microscopy.^1^ It is important to understand the acquisition of the canine AEP because of its role in plaque accumulation and associated conditions of the oral cavity, such as periodontal disease. Understanding of the strata on which the bacteria adhere is vital for improving dental health for companion animals. The AEP forms a critical layer between the mineral hydroxyapatite (HA) of the teeth and the oral biofilm. HA discs are often used to simulate the tooth surface on which the AEP can form. This allows for consistency in experimental protocols. Proteins will bind to both the negative phosphate and positive calcium sites on the HA surface. In human saliva, several phosphorylated and negatively charged saliva proteins, such as statherin, histatin 1 and acidic proline rich proteins (PRPs), bind the positive sites in HA and are considered pellicle precursor proteins.^5^ Other proteins such as epithelial derived elongation factor and myosin 9 bind negative phosphate sites. After these initial surface sites are filled protein-protein interactions play a key role in formation of subsequent layers of AEP.

Proteomic techniques have been used to identify proteins in human AEP. Initial efforts using 2-dimensional gel electrophoresis,^6^ examining AEP on HA discs, identified 200 spots but not all were sequenced. Subsequent efforts utilising Liquid-Chromatography Mass Spectrometry (LC-MS) techniques,^7^ found 130 proteins, with 89 present across multiple experiments. Fourteen percent of the proteins identified were derived from exocrine salivary secretions; the remaining 86% were derived from non-exocrine sources (eg epithelial cells and serum). The authors divided the proteins into three groups dependent on their binding efficiency to: calcium ions; phosphate ions; and other salivary proteins through protein-protein interactions.

In addition to *in vitro* examination of AEP formation, it is possible to explore the formation of AEP *in vivo*. Studies suggest that temporally AEP has high intra- and inter-individual reproducibility in humans.^8^ Samples of AEP can be taken from volunteers at given time points following a dental prophylaxis. One study examined 7 adult volunteers at 5 minutes, 10 minutes, 1 hour and 2 hours after prophylaxis and demonstrated that approximately 30% of the proteins were calcium or phosphate binding with the majority of the remainder displaying protein-protein interaction properties.^9^ The variation in the presence of eight proteins in the *in vivo* AEP time course samples was reported^9^ and suggests considerable variation per protein and per time point, with some proteins appearing to change quantity considerably (eg Mucin 5B). Additionally, a recent qualitative proteomic exploration of AEP sampled *in vivo* from children (1.5-4.5 years) has shown differences in AEP when compared to permanent teeth.^10^

The aim of this work was to explore the protein composition of canine salivary AEP formed on HA discs with qualitative and quantitative mass spectrometry techniques.

## Methods

### Canine Saliva

Sample collections described in this study were approved by The WALTHAM Petcare Science Institute Animal Welfare and Ethical Review Body. ARRIVE guidelines for pre-clinical studies were followed. A total of 10 Labrador retrievers (3 males, 7 females, aged 2.5-6.5 years) were enrolled in the study. The dogs were owned by WALTHAM and were housed by WALTHAM in kennels that exceeded the requirements of the Animal (Scientific Procedures) Act 1986 Code of Practice. Animals received tooth brushing weekly using an adult size medium bristled toothbrush and water, brushing buccal surfaces of all teeth. An oral health examination was carried out prior to the start of the trial to ensure all dogs had clinically healthy mouths. Biannual clinical examinations were routinely carried out by a veterinarian to ensure the population had no more than mild gingivitis and no sign of gingival recession. All dogs received extensive training to ensure they were relaxed, responsive, and comfortable with the sample collection procedure. Dogs were excluded from the study if they had: (1) Significant oral disease; (2) Systemic or oral antibiotic treatment, or (3) Evidence of any extraoral bacterial infections.

Saliva was collected^a^ from Labrador retriever dogs and eluted according to the manufacturer's instructions.^11^ The swab was used to sweep inside the mouth for 30 seconds to collect any pooled saliva. Sample collection took place at approximately 8 am in the morning before the morning feed. Dogs had no access to water for at least 10 min before the sample collection. The collected samples were placed on ice and immediately centrifuged at 12 000 ×g for 10 min at 4 °C then stored at −80 °C until the analyses. All samples were confirmed to contain no evidence of any blood or food material. Saliva samples were pooled from a cohort of Labrador retriever dogs to limit intra-individual variation and allow for a large enough sample for multiple experiments.

### Preparation of Hydroxyapatite Discs

Hydroxyapatite discs^b^ (diameter 10 mm, height 2 mm) were prepared for experiments by sonication in 150 µL 80% acetonitrile (ACN), 0.1% formic acid (FA) for 5 mins. The process was repeated three times replacing the 80% ACN, 0.1% FA on each repetition. After sonication, discs were rinsed with deionised H_2_O to remove excess ACN.

### Pellicle Formation on Hydroxyapatite Discs

Hydroxyapatite discs were submerged in 150 µL pooled, filtered, Labrador retriever saliva (1 mg/mL) for up to 1 hour at 38 °C. Discs were then washed in deionised H_2_0 and sonicated in 80% ACN, 0.1% FA for 5 mins, for a total of three times, in a water bath sonicator^c^. For each sonication, the 80% ACN, 0.1% FA was aspirated from the discs and replaced. The aspirated eluents were pooled together for each disc and concentrated to 10 µL in a vacuum centrifuge^d^. Samples were then diluted to a final volume of 100 µL with triethylammonium bicarbonate (TEAB, 100 mM) and used in subsequent experiments.

### Preparation of Samples for LC-MS Analysis

#### Protein assay

Using the Pierce bicinchoninic acid (BCA) protein assay kit^e^ 10 µL of the bovine serum albumin standards (0.025 to 2 mg/mL) and the unknown saliva samples were dispensed into a 96 well microplate. BCA reagent was made up according to the manufacturer's instructions and 200 µL was dispersed to each well containing the standards and unknown samples. The plate was incubated at 37 °C for 30 mins, cooled to room temperature and the absorbance read at 570 nm^f^.

#### Digestion

Protein (30-100 µg) was made to a final volume of 100 µL in triethylammonium bicarbonate (TEAB). Samples were reduced with tris(2-carboxyethyl)phosphine hydrochloride (TCEP) at 55 °C for 1 hour and alkylated with iodoacetamide at room temperature for 30 mins in the dark. The alkylation reaction was quenched with the addition of dithiothreitol (50 mM) and trypsin^g^ was added at 1:50 (trypsin: protein). Protein samples were digested overnight at 37 °C.

#### Tandem mass tag (TMT)10plex labelling

Digested peptides were labelled with tandem mass tags (TMT10plex)^e^ following manufacturer's instructions.

#### Mass spectrometry

Liquid chromatography: 1.5 µg of peptides were loaded on to a 150 mm Acclaim PepMap100 C18 column^e^ in formic acid (0.1% v/v, mobile phase A). Peptides were separated over a linear gradient from 3.2% to 44% mobile phase B (acetonitrile with formic acid (0.1% v/v)) with a flow rate of 350 nL/min. The column was then washed with 90% mobile phase B before re-equilibrating at 3.2% mobile phase B. The column oven was heated to 35 °C. The high-performance liquid chromatography system^h^(LC) system was coupled to an orbitrap mass spectrometer^i^ which infused the peptides directly into an linear trap quadrupole (LTQ)-Orbitrap electron-transfer dissociation (ETD)^e^.

Proteomic analysis: The mass spectrometer performed a full fourier transform (FT)-MS scan (m/z 380-1800) and subsequent collision induceddissociationMS/MS scans of the 7 most abundant ions above an absolute signal intensity threshold of 5000 counts. Full scan mass spectra were recorded at a resolution of 60 000 at m/z 400 and automatic gain control (ACG) target of 1 × 10^6^ (maximum injection time 1 sec). Precursor ions were fragmented in collision induced dissociation (CID) MS/MS with a normalised collision energy of 35% and an activation Q of 0.25. ACG target for CID MS/MS was 1 × 10^5^ (maximum injection time 50 ms). The width of the precursor isolation window was 2 m/z and only multiply-charged precursor ions were selected for MS/MS. Spectra were acquired for 56 mins. Data are reported as peptide spectral matches (PSMs).

Quantitative analysis: A full FT-MS scan (m/z 380-1800) was performed with subsequent higher-energy C-trapdissociation (HCD) MS/MS scans of the 7 most abundant ions that passed a minimum signal requirement of 5000 counts. The full FT-MS scans were recorded at 120 000 resolution and ACG target of 1 × 10^6^ (maximum injection time 1 sec). Precursor ions were fragmented in HCD MS/MS with a normalised collision energy of 38% and an activation time of 0.1. ACG target for HCD MS/MS was 1 × 10^5^ (maximum injection time 50 ms). The width of the precursor isolation window was 2 m/z and only multiply-charged precursor ions were selected for MS/MS. FT first mass value was reduced to 120 m/z to account for TMT reporter ions and spectra were acquired for a total of 88 mins. Data are reported as ratios to time 0.05 min.

#### Bioinformatics

**Figure 1.**
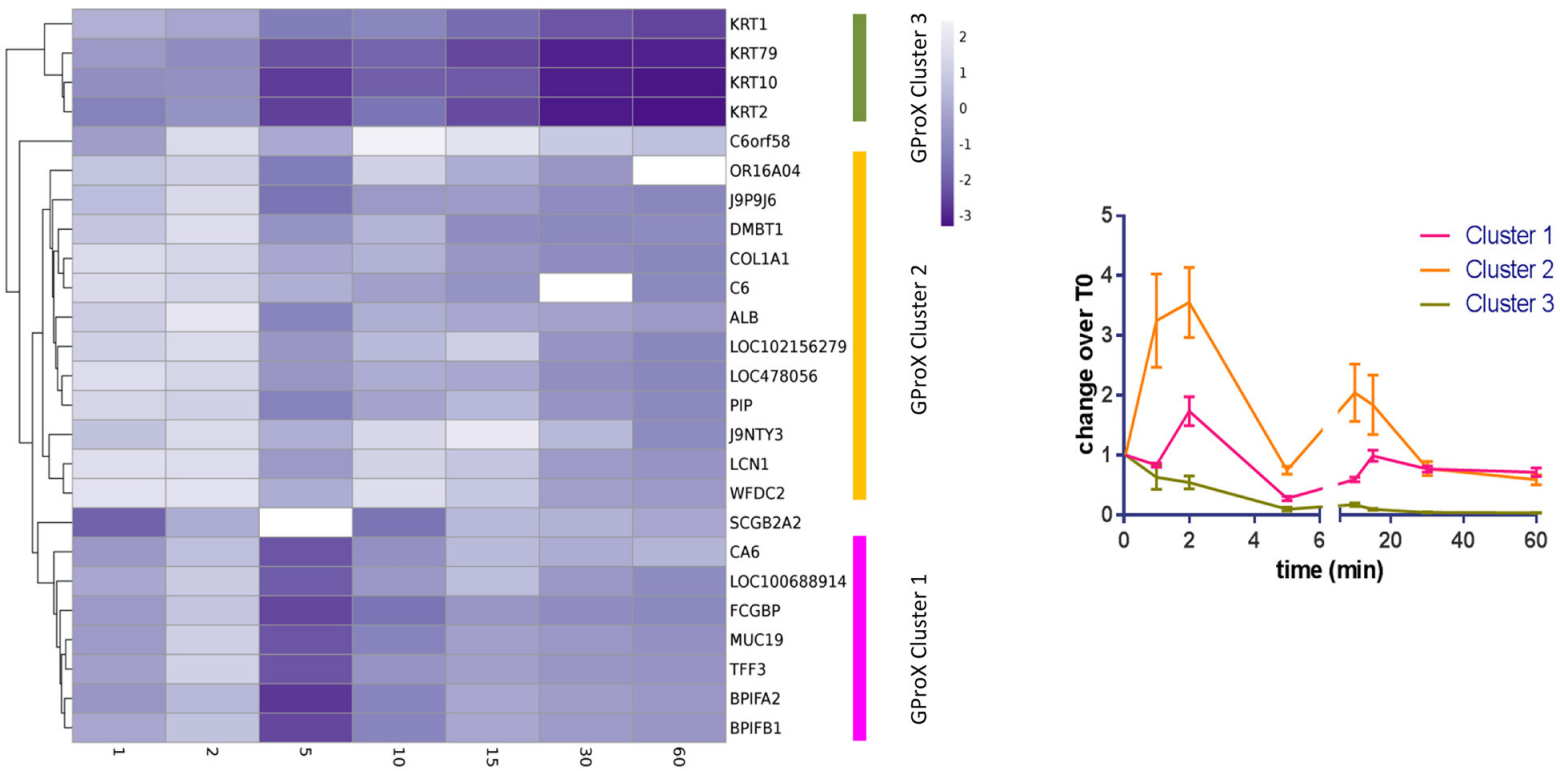
Cluster analysis of the protein profiles from TMT analysis. Left diagram. Heatmap showing abundance of proteins at different times. Three clusters were estimated and are marked on the right hand side. Missing values are shown in white. Right diagram. Visualisation of the cluster profiles. Mean ± standard error of the mean shown.

MS raw data files were analysed^j^ to identify the proteins present in each sample in a qualitative analysis. Both Sequest and Mascot were used to search against the *Canis lupus familiaris* database (a combination of SwissProt and TrEMBL databases). Carbamidomethylation of cysteine was added as a fixed modification and deamidation of asparagine and glutamine and oxidation of methionine were added as variable modifications to the searches. Search results were filtered with a 1% false discovery rate. Quantitative analysis was performed^k^ by means of the same fixed and variable modifications as in the qualitative analysis. Relative abundance was considered significant if a minimum of 2-fold increase or decrease was demonstrated. Subsequent clustering and gene ontology analysis were performed^l^. For cluster analysis data were log 2 transformed and visualised using Euclidean distance and average linkage. Nipals principal component analysis was used for missing value estimation (Figure 1). Where gene IDs were unavailable BLAST searches were employed to determine the putative protein identification in the human proteome.

## Results

Initial experiments sought to identify proteins in the AEP after incubation of HA discs in pooled canine saliva for 1 hour. Ninety-six proteins were identified with at least 2 peptides in at least one experiment (Table 1). Thirty-four proteins were identified in all five experiments, which likely indicate the most abundant proteins in canine AEP at 1 hour. This level of overlap between experiments is expected in qualitative experimentation. It was possible to suggest that 11% of the proteins detected may bind calcium, 3% may bind phosphate and 39% may have protein-protein interaction partners when compared to a previous study.^9^ Comparison of the proteins listed in Table 1 with the International Molecular Exchange (IMEx) Database for protein-protein interactions^12^ estimated 80% of the proteins detected may have protein-protein interaction partners. With either estimate of protein-protein interaction, it is implied that there could be many proteins brought to the pellicle without direct interaction with the surface. The five proteins identified with the most peptide spectral matches were Mucin 19 (MUC19), Fc fragment of IgG binding protein (FCGBP), bactericidal/permeability-increasing (BPI) fold containing family A member 2 (BPIFA2), BPI fold containing family B member 1 (BPIFB1) and carbonic anhydrase 6 (CA6). FCGBP, BPIFA2 and BPIFB1 may all demonstrate antimicrobial action either by binding antibodies or by providing antimicrobial peptides.

**Table 1.** Proteins Identified in Extracted AEP Formed After 1 Hour Using a Qualitative Analysis of 5 Independent Experiments. Duplicate Entries with Different Accession Numbers Have Both Been Reported. The Latter Are Identified as N/A (Not Available) and Putative. Bold Indicates Proteins Previously Found in Human AEP.^9^

Accession	Number of times identified	Median coverage	Median PSMs	Gene name	Description
E2QWJ3	5	58.11	80	BPIFB1	BPI fold containing family B, member 1
E2QXJ0	5	53.82	90	BPIFA2	BPI fold containing family A, member 2
E2QYS2;H2B3G5	5	34.74	22	Cfa.21327	Lipocalin-Can f 6 allergen
**E2R0H6**	**5**	**62.07**	**27**	**PIP**	**prolactin-induced protein**
E2R2B9	5	12.88	7	BPIL1	BPI fold containing family B, member 2
E2RD02	5	33.01	26	C6orf58	chromosome 1 open reading frame, human C6orf58
**E2RFI9**	**5**	**27.3**	**40**	**LPO**	**Lactoperoxidase**
E2RH46	5	33.4	137	FCGBP	Fc fragment of IgG binding protein
F1P6A9	5	59.14	186	MUC19	Mucin 19
F1P931	5	40.52	12	LOC607055	angiopoietin-related protein 5-like
F1PBS8	5	49.43	27	LCN1	lipocalin 1
**F1PCG9**	**5**	**50.31**	**66**	**CA6**	**Carbonic anhydrase 6**
F1PPU5	5	52.5	14	TFF3	Trefoil factor 3
**F1PQL8**	**5**	**19.84**	**12**	**ACTG1**	**actin, gamma 1**
F1PR78	5	81.74	59	LOC478056	double-headed protease inhibitor, submandibular gland-like
F1PSX9	5	12.85	10	HYAL1	hyaluronoglucosaminidase 1
F1PTS8	5	13.13	12	KRT6A	keratin 6A
F1PTY1	5	10.5	13	KRT1	keratin 1
**F1PYU9**	**5**	**24.3**	**41**	**KRT10**	**Keratin, type I cytoskeletal 10**
F1Q0R0	5	10.58	12	KRT16	keratin 16
F1Q2Y5	5	12.7	3	ZG16B	zymogen granule protein 16B
**F2Z4Q6**	**5**	**39.8**	**51**	**ALB**	**Serum albumin**
F6USN4	5	24.66	66	LOC611455	ovostatin homologue 2-like
**F6Y6T8**	**5**	**19.42**	**26**	**PIGR**	**polymeric immunoglobulin receptor**
**G1K265**	**5**	**32.43**	**8**	**LYZ**	**Lysozyme**
J9NSZ4	5	15.84	18	LOC102156279	submaxillary mucin-like protein-like
J9NTI8	5	54.95	9	LOC100688914	secretoglobin family 1D member 2-like
J9NUN7	5	59.14	15	N/A	N/A (putative: Mammaglobin-A)
J9NVT1	5	21	2	LOC609896	ubiquitin-40S ribosomal protein S27a-like
**J9NWS3**	**5**	**2.69**	**3**	**KRT9**	**keratin 9**
**J9P2K8**	**5**	**7.31**	**6**	**MUC7**	**Mucin 7**
J9P839	5	41.98	16	SCGB2A2	secretoglobin, family 2A, member 2
O18874	5	74.44	19	N/A	Minor allergen Can f 2
P01002	5	80	25	N/A	double-headed protease inhibitor, submandibular gland
E2QUN9	4	36.415	4.5	N/A	N/A (putative: Major allergen I polypeptide chain 1)
E2QWD8	4	8.23	9	N/A	N/A (putative: Vomeromodulin)
E2R7W6	4	7.615	9	KRT14	keratin 14
E2R8Z5	4	10.905	12.5	KRT5	keratin 5
**E2RLH6**	**4**	**53.29**	**16**	**LOC480784**	**hemoglobin subunit beta-like**
E2RPK8	4	20.59	7	PEBP4	phosphatidylethanolamine-binding protein 4
**F1PCH3**	**4**	**3.03**	**2**	**ENO1**	**enolase 1, (alpha)**
F1PHY1	4	1.87	3.5	COL1A2	Collagen alpha-2(I) chain
**F1PR54**	**4**	**2.54**	**4**	**LTF**	**Lactotransferrin**
F1PSX2	4	50.945	9	N/A	N/A (putative: Ig lambda-2 chain C regions)
**F1PTX4**	**4**	**10.08**	**17**	**KRT2**	**Keratin, type II cytoskeletal 2**
F1PW98	4	3.65	3	KRT8	keratin 8
F1Q3I5	4	2.22	4.5	COL1A1	Collagen alpha-1(I) chain
J9JHH5	4	22.01	6	IGJ	immunoglobulin J polypeptide, linker protein for immunoglobulin alpha and mu polypeptides
J9P9J6	4	27.365	18.5	N/A	N/A (putative Immunoglobulin heavy chain variant)
**L7N0D6**	**4**	**8.94**	**8.5**	**DMBT1**	**deleted in malignant brain tumours 1**
A1ILJ0	3	23.13	10	N/A	Alpha 1 antitrypsin
**C0LQL0**	**3**	**41.57**	**16**	**Cfa.42417**	**S100 calcium binding protein A8**
E2QXE7	3	12.16	5	BPIFA1	BPI fold containing family A, member 1
E2RKJ6	3	7.67	5	SERPINB9	serpin peptidase inhibitor, clade B (ovalbumin), member 9
F1P9E5	3	2.7	2	PLA2G7	phospholipase A2, group VII (platelet-activating factor acetylhydrolase, plasma)
**F1PDJ7**	**3**	**26.98**	**14**	**AZGP1**	**alpha-2-glycoprotein 1, zinc-binding**
F1PDT8	3	40.29	9	WFDC2	WAP four-disulfide core domain 2
**J9NS29**	**3**	**29.41**	**6**	**LOC607874**	**cystatin-C-like**
J9P7B6	3	54.95	25	LOC100687441	secretoglobin family 1D member-like
J9PAQ5	3	58.7	9	S100A12	S100 calcium binding protein A12
L7N0F2	3	56.35	15	LOC486474	immunoglobulin lambda-like polypeptide 5-like
**Q29474**	**3**	**16.48**	**6**	**N/A**	**Kallikrein**
Q8MJD1	3	6.03	2	ELA2	Neutrophil elastase
**A0A077S9R2**	**2**	**29.05**	**9**	**LYZF2**	**Lysozyme**
E2QUP2	2	20.18	6	LOC102153923	major allergen I polypeptide chain 2-like
**E2R5U8**	**2**	**32.65**	**7**	**TTR**	**Transthyretin**
E2R5W6	2	6.96	7	GC	group-specific component (vitamin D binding protein)
**E2R7U2**	**2**	**10.385**	**10.5**	**KRT13**	**Keratin 13**
E2R917	2	7.7	7.5	KRT75	Keratin 75
E2RB38	2	8.06	4	TPM1	tropomyosin 1
E2RJE4	2	26.17	6	CST6	cystatin E/M
E2RLF1	2	12.02	12	PSAP	Prosaposin
E2RN09;E2RN10	2	16.8	4	B2M	Beta-2-microglobulin
F1PBL1	2	11.43	6	YWHAZ	tyrosine 3-monooxygenase/tryptophan 5-monooxygenase activation protein, zeta polypeptide
**F1PDJ5**	**2**	**34.21**	**13**	**APOA1**	**apolipoprotein A-I**
F1PQ93	2	33.06	13	SFN	Stratifin
F1Q0B9	2	76.14	44	KLK1	kallikrein 1
F1Q3X2;F1PS73	2	42.86	3	CSTB	Cystatin-B
F1Q462	2	14.67	3	SOD1	Superoxide dismutase [Cu-Zn]
**F2Z4N8**	**2**	**17.02**	**13**	**ACTG2**	**actin, gamma 2**
**G1K2D9**	**2**	**10.09**	**6**	**LOC479668**	**haptoglobin-like**
J9JHF7	2	57.04	16	LOC100855540	hemoglobin subunit alpha-like
J9NXL3	2	57.04	16	LOC100855558	hemoglobin subunit alpha-like
J9NYW7	2	6.7	3	N/A	N/A (putative: Ig gamma-1 chain C region)
J9P430	2	8.6	12	TF	Transferrin
J9P950	2	31.61	7	OBP	allergen Can f 4
J9P9H8	2	19.71	2.5	LOC100855558	Hemoglobin subunit alpha
O77704	2	3.07	3	DSC2	desmocollin 2
P00011	2	23.81	2	CYCS	Cytochrome c
**P49822**	**2**	**65.46**	**59**	**ALB**	**Serum albumin**
**P60524**	**2**	**78.08**	**13**	**HBB**	**hemoglobin subunit beta**
E2RCC8	1	5.29	3	IGHM	IgG H chain
F1PBZ4	1	13.09	5	NQ01	NAD(P)H dehydrogenase [quinone] 1
F1Q0Q9	1	17.55	13	KRT17	Keratin 17
J9P9J6	1	35.5	28	N/A	N/A (putative: Ig alpha chain C region)
L7N097	1	5.96	4	KRT7	Keratin 75

Abbreviations: ALB, albumin; BPI, bactericidal/permeability-increasing; CSTB, cystatin B; CYCS, cytochrome c; HBB, haemoglobin beta; IGHM, immunoglobulin H chain; IGJ, immunoglobulin J chain; LYZ, lysozyme; NAD(P)H, nicotinamide adenine dinucleotide phosphate; OBP, odorant-binding protein; PSAP, prosaposin; SFN, stratifin; WAP, whey-acidicprotein; YWHAZ, tyrosine 3-monooxygenase/tryptophan 5-monooxygenase activation protein, zeta polypeptide.

Next, protein adsorption to HA discs over a 1-hour time course was assessed using quantitative mass spectrometry methods. Proteins bound to the HA discs were eluted at several time-points and subsequently digested. The proteomic profiles of the AEP were compared and demonstrated changes that occur to the pellicle over time (Table 2, Figure 1).

**Table 2.** Proteins Identified During AEP Formation By Quantitative Mass Spectrometry. Data is Shown as Relative Abundance in Comparison to 0.05 min. Duplicate Entries With Different Accession Numbers Have Both Been Reported. The Latter Are Identified As N/A (Not Available) And Putative. Nd Represents Not Detected. Bold Indicates Proteins Previously Found in Human AEP.^9^

Accession	gene name	Description	0.05 min	1 min	2 min	5 min	10 min	15 min	30 min	60 min	Cluster	Potential binding
F1P6A9	MUC19	Mucin 19	1	0.8	1.81	0.31	0.54	0.87	0.75	0.67	1	Protein-protein interactions
E2QXJ0	BPIFA2	BPI fold containing family A, member 2	1	0.72	1.28	0.19	0.51	0.96	0.80	0.74	1	
F1PR78	LOC478056	double-headed protease inhibitor, submandibular gland-like	1	4.73	3.77	0.56	1.04	0.96	0.44	0.35	2	
E2QWJ3	BPIFB1	BPI fold containing family B, member 1	1	0.97	1.47	0.26	0.56	0.96	0.81	0.73	1	
E2RH46	FCGBP	Fc fragment of IgG binding protein	1	0.82	1.41	0.35	0.52	0.79	0.69	0.66	1	Protein-protein interactions
F1PBS8	LCN1	lipocalin 1	1	3.21	2.92	0.72	2.33	1.75	0.77	0.63	2	
**F1PTY1**	**KRT1**	**keratin 1**	**1**	**1.22**	**0.86**	**0.18**	**0.26**	**0.11**	**0.06**	**0.04**	**3**	**Protein-protein interactions**
**E2R0H6**	**PIP**	**prolactin-induced protein**	**1**	**1.82**	**1.69**	**0.58**	**0.93**	**1.21**	**0.74**	**0.64**	**2**	**Protein-protein interactions**
**F2Z4Q6**	**ALB**	**Serum albumin**	**1**	**1.49**	**2.10**	**0.66**	**1.04**	**0.97**	**0.92**	**0.85**	**2**	**Protein-protein interactions**
J9NSZ4	LOC102156279	submaxillary mucin-like protein-like	1	2.61	3.52	0.61	1.46	2.49	0.59	0.41	2	
**F1PCG9**	**CA6**	**Carbonic anhydrase 6**	**1**	**0.82**	**1.27**	**0.44**	**0.76**	**1.19**	**1.01**	**1.11**	**1**	**Calcium and phosphate**
F1PDT8	WFDC2	WAP four-disulfide core domain 2	1	7.58	7.70	1.05	6.07	2.63	0.70	0.59	2	
F1PVL5	KRT79	Keratin 79	1	0.59	0.36	0.07	0.11	0.06	0.03	0.03	3	
J9NTY3		N/A (putative WAP four-disulfide core domain protein 3)	1	1.86	3.79	1.12	3.40	6.24	1.45	0.43	2	
J9P9J6		N/A (putative Immunoglobulin heavy chain variant)	1	1.34	2.18	0.43	0.76	0.81	0.63	0.56	2	
A9Q6H6	TFF3	Trefoil factor family peptide 3	1	0.77	3.10	0.11	0.55	0.74	0.56	0.54	1	
**F1PYU9**	**KRT10**	**Keratin, type I cytoskeletal 10**	**1**	**0.46**	**0.49**	**0.08**	**0.15**	**0.14**	**0.05**	**0.05**	**3**	**Protein-protein interactions**
F1Q3I5	COL1A1	Collagen alpha-1(I) chain	1	8.44	6.58	0.90	1.36	0.44	0.28	0.22	2	
**F1PTX4**	**KRT2**	**Keratin, type II cytoskeletal 2**	**1**	**0.26**	**0.47**	**0.05**	**0.17**	**0.07**	**0.03**	**0.02**	**3**	**Protein-protein interactions**
Q70Z96	OR16A04	Olfactory receptor	1	2.28	3.47	0.21	3.79	1.07	0.52		2	
J9NTI8	LOC100688914	secretoglobin family 1D member 2-like	1	0.96	1.82	0.28	0.74	1.43	0.73	0.56	1	
J9P839	SCGB2A2	secretoglobin, family 2A, member 2	1	0.40	1.02		0.48	1.20	1.12	0.94	-	
E2RD02	C6orf58	chromosome 1 open reading frame, human C6orf58	1	0.85	1.88	1.00	2.85	2.20	1.50	1.30	-	
**L7N0D6**	**DMBT1**	**deleted in malignant brain tumours 1**	**1**	**1.79**	**2.94**	**0.61**	**1.23**	**0.54**	**0.51**	**0.52**	**2**	**Protein-protein interactions**
E2RGT6	C6	NA (putative Complement component C6)	1	2.51	2.29	1.06	0.84	0.64	nd	0.52	2	

To determine whether proteins shared similarities in adsorption patterns based on changes over time, the plots were clustered (Figure 1). Three cluster groups, with two outliers, were produced demonstrating different patterns of adsorption and desorption: cluster 1 shows a minor biphasic pattern; cluster 2 a more pronounced biphasic pattern; and cluster 3 shows a decrease in protein relative abundance across the time points compared to the start. Cluster 1 is dominated by proteins of salivary origin, which are known to be highly abundant. Cluster 2 contains proteins of cellular, salivary and plasma origins. By the end of the hour of pellicle formation both cluster 1 and cluster 2 proteins appear to be of a similar quantity relative to the start. Cluster 3 is composed of four proteins, all of which are keratins. The source of these proteins is likely to be from the oral cavity itself.

## Discussion

This study has demonstrated that we can analyse AEP proteins from canine saliva by mass spectrometry using qualitative and quantitative methods. The temporal experiments show that the AEP forms extremely rapidly and is stable over an hour. Our data demonstrates the initial binding occurring within a few seconds and the equilibration of protein adsorption after 30 minutes. This pattern mimics what is reported about the two phases that occur in pellicle formation in humans: the initial phase and secondary phase. In the initial phase precursor proteins adhere to the tooth enamel within seconds of exposure to saliva.^13^ This is followed by the secondary phase between 30 to 90 minutes after saliva exposure in which protein aggregates bind and a plateau is reached in pellicle formation.^14^ This could be explained by the Vroman effect^15^ whereby the most mobile proteins arrive at a surface first but these are later replaced by less mobile proteins that have higher affinity for the surface. It has been suggested^2,16^ that the later proteins may be in the form of protein aggregates, which would be less mobile or may be modified by enzymatic acction.^17^ In this study it is possible to see that there are changes to individual protein patterns indicating how they change at the surface. This demonstrates a specific dynamic alteration of the protein species in the formation of AEP.

The data presented here has some similarities with that published whereby the authors demonstrate different patterns of protein adsorption across a 2 hour *in vivo* experiment.^9^ Our experiments are different due to the earlier time points used which give more focus on the initial changes in pellicle formation. Previous studies suggest that during these early time points there is salivary protein adsorption, particularly by isolated proteins rather than agglomerates which adhere later.^16,18^ Here we have highlighted similar proteins to those reported previously for human pellicle formation as studied by mass spectrometry.^9^ As dogs do not express amylase^19^ this is not reported, nonetheless we show data for Mucins 7 and 19, lactoperoxidase and lysozyme. In contrast, proteomic analysis of human saliva readily finds histatins and statherin, however, it was not possible to find these proteins in our canine AEP data set although they are reported by another study^9^ to be components of the human AEP. Published data from studies examining canine saliva have not reported these two proteins.^20–22^

Furthermore, comparison to proteins detected in canine saliva^21,22^ suggests that a range of proteins including the most abundant are forming the AEP. Using peptide spectral matches as a crude ranking of abundance in the canine saliva proteome and the canine AEP shows that the top 15 proteins found in the saliva dataset are found in five qualitative replicates present here. BPI fold containing proteins, BPIFA2 and BPIFB1, MUC19, Fc fragment of IgG binding protein (FCGBP) and carbonic anhydrase are particularly abundant in both saliva and the pellicle. They form cluster 1 which displays a less pronounced biphasic pattern in the quantitative experiment, suggesting these proteins may have a more stable presence or interaction with hydroxyapatite. Cluster 1 also contained carbonic anhydrase which buffers saliva and is functional in AEP, in humans it is suggested that it may help in the prevention of dental caries by direct surface acid neutralisatio.^23^ In cluster 2 fewer proteins were estimated at such high abundance and the magnitude of the change in presence across the quantitative time course was greater, suggesting that these proteins are more dynamic or mobile in their interaction, although there appeared to be no appreciable or statistical (T-test) difference in the molecular weight or isoelectric point for proteins in clusters 1 or 2. Cluster 2 did appear to contain a number of protease inhibitors such as LOC478056 (double headed protease inhibitor), J9NTY3, lipocalin and whey-acidicprotein four-disulphide core domain 2 (WFDC2), potentially forming a subcluster, although their role is unknown. In cluster 3 there are four keratins of epithelial origin that decrease within the time course. Keratins are found naturally in the oral cavity and are regularly found within saliva samples^21^ and have been found in human AEP.^9^ Oral epithelial cells, the most likely source, are continuously desquamating to aid in removal of the oral microflora. It may be that they are brought to the surface through a weak protein-protein interaction and are lost from the surface through temporal changes resulting in the slow decline over the observed time.

Lastly there were two proteins which appeared to lie outside of the three main clusters: C6orf58 and secretoglobin family 2A member 2 (SCGB2A2). C6orf58 is a protein LEG1 homologue which has previously been found in human saliva and in hypomineralized dental enamel and is known to be glycosylated; other properties and functions do not appear to be defined. SCGB2A2 is also glycosylated and has been investigated for its role in breast cancer. Limited information is known about its role in saliva.

This study was conducted using an *in vitro* model of HA discs. These discs represent the tooth surface however will not experience the shear forces as if they were present in the mouth or if enamel itself were used. *In vivo* studies utilising dental stents to track AEP development *in situ* are challenging in companion animals for both ethical and practical reasons.

In summary we have demonstrated that canine saliva will form a pellicle on hydroxyapatite surfaces, the pellicle is evident from very early time points, and that the protein content can be determined through proteomic analysis. Highly abundant salivary proteins are prominent and there is a biphasic profile to the acquisition of the pellicle over one hour of observation, which may be reflective of the Vroman effect; the movement of isolated proteins that are then potentially followed by protein complexes or agglomerates. Proteins adhered to the HA surface display calcium, phosphate and protein binding capabilities and could form the basis for bacterial adhesion to a tooth surface. A deeper understanding of the processes leading to bacterial colonisation of the canine tooth surface will lead to a clearer picture of the driving factors behind dental plaque accumulation.

## Materials

- SalivaBio Children's Swabs, Salimetrics, USA
- Clarkson Chromatography, South Williamsport, USA
- VWR, UK
- Eppendorf, UK
- Thermo Fisher Scientific, UK
- Infinite f260pro spectrometer, Tecan, UK
- Promega, UK
- 3000 Ultimate, Dionex, UK
- LTQ-Orbitrap Elite ETD, Thermo Fisher Scientific, UK
- Proteome Discoverer 1.4, Thermo Fisher Scientific, UK
- MaxQuant v.1.5, Max Planck Institute of Biochemistry, Germany
- GProX v.1.1, Centre for Experimental BioInformatics, University of Southern Denmark and PANTHER Thomas Lab, University of South California USA
